# Casein Phosphopeptide-Amorphous Calcium Phosphate Reduces *Streptococcus mutans* Biofilm Development on Glass Ionomer Cement and Disrupts Established Biofilms

**DOI:** 10.1371/journal.pone.0162322

**Published:** 2016-09-02

**Authors:** Stuart G. Dashper, Deanne V. Catmull, Sze-Wei Liu, Helen Myroforidis, Ilya Zalizniak, Joseph E. A. Palamara, N. Laila Huq, Eric C. Reynolds

**Affiliations:** Oral Health Cooperative Research Centre, Melbourne Dental School, Bio21 Institute, The University of Melbourne, Melbourne, Australia; Laurentian, CANADA

## Abstract

Glass ionomer cements (GIC) are dental restorative materials that are suitable for modification to help prevent dental plaque (biofilm) formation. The aim of this study was to determine the effects of incorporating casein phosphopeptide-amorphous calcium phosphate (CPP-ACP) into a GIC on the colonisation and establishment of *Streptococcus mutans* biofilms and the effects of aqueous CPP-ACP on established *S mutans* biofilms. *S*. *mutans* biofilms were either established in flow cells before a single ten min exposure to 1% w/v CPP-ACP treatment or cultured in static wells or flow cells with either GIC or GIC containing 3% w/w CPP-ACP as the substratum. The biofilms were then visualised using confocal laser scanning microscopy after BacLight LIVE/DEAD staining. A significant decrease in biovolume and average thickness of *S*. *mutans* biofilms was observed in both static and flow cell assays when 3% CPP-ACP was incorporated into the GIC substratum. A single ten min treatment with aqueous 1% CPP-ACP resulted in a 58% decrease in biofilm biomass and thickness of established *S*. *mutans* biofilms grown in a flow cell. The treatment also significantly altered the structure of these biofilms compared with controls. The incorporation of 3% CPP-ACP into GIC significantly reduced *S*. *mutans* biofilm development indicating another potential anticariogenic mechanism of this material. Additionally aqueous CPP-ACP disrupted established *S*. *mutans* biofilms. The use of CPP-ACP containing GIC combined with regular CPP-ACP treatment may lower *S*. *mutans* challenge.

## Introduction

Dental caries is one of the most prevalent chronic diseases of humans and affects the majority of individuals globally [1]. The disease is a dynamic process that is initiated in the bacterial biofilm (dental plaque) on the tooth surface by the production of organic acids from the fermentation of dietary sugar resulting in demineralisation of the tooth [2]. Caries is a multifactorial process that requires a shift in plaque ecology to favour acidogenic and aciduric microbial species that is usually driven by frequent consumption of simple carbohydrates and is modified by host factors such as saliva [3]. Although dental caries has a polymicrobial aetiology *Streptococcus mutans* is regarded as a major aetiological agent of this disease mainly due to its aciduric and highly acidogenic nature.

Casein phosphopeptide-stabilised amorphous calcium phosphate (CPP-ACP) acts as a salivary biomimetic that provides calcium and phosphate ions in a bioavailable form allowing these ions to inhibit demineralisation and promote the remineralisation of early caries lesions [4, 5]. The remineralisation of enamel subsurface lesions by CPP-ACP, mainly in the form of an applied tooth creme, has been demonstrated in numerous *in vitro*, *in situ* and *in vivo* studies [6–16]. In addition CPP-ACP incorporated into a sugar-free gum has been shown to slow the progression and enhance the regression of dental caries in a randomised, controlled clinical trial [17].

Studies on the mechanisms of action of CPP-ACP have focussed on its provision of bioavailable calcium and phosphate ions and its remineralisation capabilities however there have also been some reports of its ability to bind to *S*. *mutans* and inhibit adhesion to the tooth surface [18–20]. In addition two recent clinical studies demonstrated that CPP-ACP reduced *S*. *mutans* burden. Once daily treatment of children from 18 months to 24 months of age with a CPP-ACP containing tooth creme was shown to reduce recovery of *S*. *mutans* compared with the control [21]. In a clinical trial of 60 subjects, chewing CPP-ACP chewing gum three times daily resulted in a significant reduction in salivary *S*. *mutans* levels after three weeks [22]. This indicates that CPP-ACP has an inhibitory effect on *S*. *mutans*, and potentially other oral bacteria, that may contribute to its anticariogenicity *in vivo*.

Glass Ionomer Cements (GIC) have been used in a number of applications since their first introduction to the dental profession in 1972 [23]. GICs promote remineralisation of the tooth and inhibition of bacterial growth which has largely been attributed to the release of fluoride from the material [24, 25]. GC Fuji VII Enhanced Protection (EP) is a GIC containing 3% CPP-ACP (Recaldent^TM^). It was proposed that by adding CPP-ACP to this GIC there would be a greater inhibition of bacterial binding and proliferation conferring greater protection of the tooth surface at risk of developing caries [26]. Previous studies have shown that CPP-ACP addition to a conventional GIC increased compressive and microtensile bond strength, enhanced release of calcium, phosphate and fluoride ions and enhanced protection of the adjacent dentine to acid demineralisation [27].

The aim of this study was to determine the antibiofilm effects of the incorporation of CPP-ACP into GIC to inhibit the colonisation of the surface and prevent the development of *S*. *mutans* biofilms and the effects of aqueous CPP-ACP on established *S*. *mutans* biofilms.

## Materials and Methods

### Growth and culture of *S*. *mutans*

*S*. *mutans* Ingbritt was propagated in Todd-Hewitt Yeast Extract medium (36.4 g/L Todd Hewitt broth, 8 g/L of yeast extract and 1 g of sucrose/L) at 37°C from freeze-dried bacterial stocks kept at the Melbourne Dental School.

All biofilm assays were performed using a 25% Artificial Saliva Medium containing (0.625 g/L type II porcine gastric mucin, 0.5 g/L bacteriological peptone, 0.5 g/L tryptone, 0.25 g/L yeast extract, 0.088 g/L NaCl, 0.05 g/L KCl, 0.05 g/L CaCl_2_ and 1 mg/L haemin, pH 7.0; supplemented with 2.5 mM DTT and 0.5 g/L sucrose. The media was sterilised at 121°C for 15 min.

### Preparation of Fuji VII and Fuji VII EP GIC

Fuji VII and Fuji VII EP containing 3% w/w CPP-ACP from a matching batch were provided by GC Corporation (Japan) in capsule form. Polyvinyl siloxane impression material (eliteHD+ light body, Zhermack SpA, Badia Polesine, Italy) moulds were used to create standardised GIC discs measuring 10 mm diameter x 1.5 mm thickness for flow cells and blocks of 10 mm^2^ x 1.5 mm thickness for static biofilm assays. Each GIC disc was prepared by placing the materials in the mould with the top and bottom surfaces covered by plastic strips, held between two glass slides. The glass slides were pressed together to extrude excess material. The discs were allowed to set inside the moulds for 24 h in an incubator at 37°C with ~100% relative humidity. After cooling to room temperature the discs were removed from the moulds and lapped with 600 grit paper to mimic the finished restorative material applied *in vivo*. (Norton Tufbak, Saint-Gobain Abrasives Ltd., Auckland, NZ).

### Static biofilm assay with Fuji VII GIC and Fuji VII EP

A 1 mL inoculum containing 1 x 10^9^ *S*. *mutans* cells were placed into each well of a 6 well Nunclon tissue culture chamber (Nunclon, NUNC) containing 1 mL of 25% ASM and one 10 mm^2^ block of either Fuji VII or Fuji VII EP GIC (3 blocks of each type were tested per experiment). The chamber was wrapped with parafilm to prevent evaporation and incubated at 37°C for 16 h. After 16 h blocks were removed and washed in sterile distilled water to remove unbound cells and then stained with BacLight Live/Dead stain (Invitrogen) prepared as per manufacturer’s instructions for 15 min. The blocks were rinsed again to remove excess stain and placed into Coverwell imaging chambers (Invitrogen) containing custom made inserts to enable the undisturbed imaging of the biofilm. Prolong Gold anti-fade solution (Invitrogen) was then added to the block and the chamber sealed with a coverslip and clear nail varnish.

### Flow cell biofilm assay with Fuji VII GIC and Fuji VII EP

**Fig 1:**
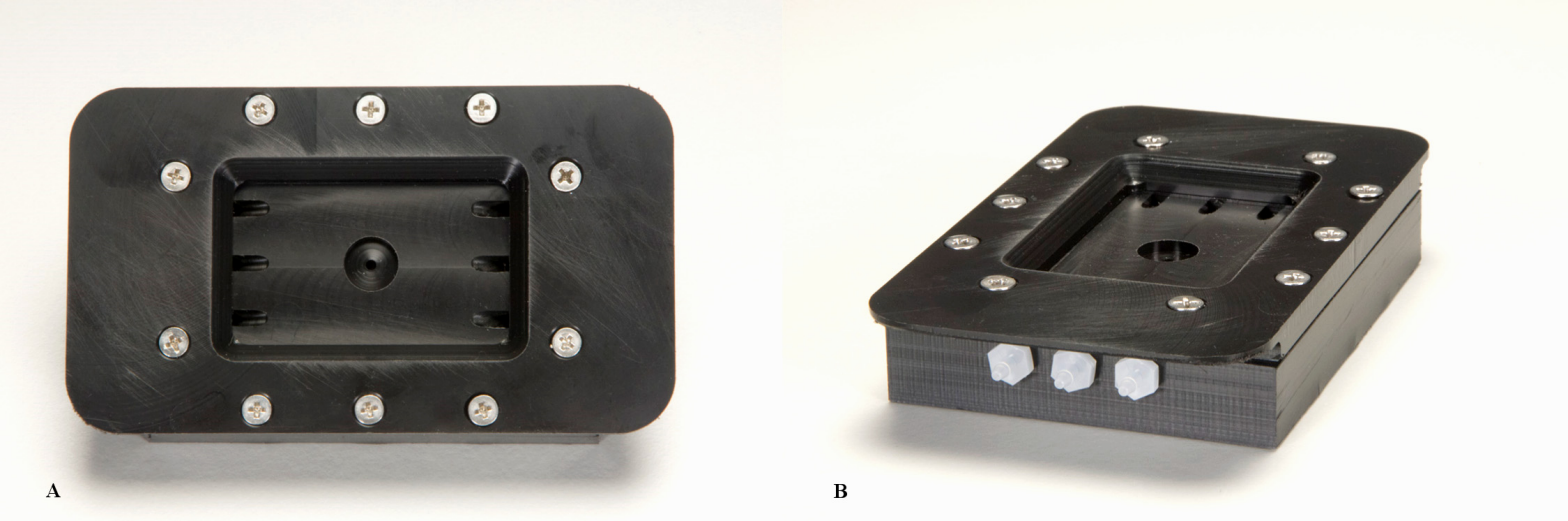
Three channel flow cell system for the visualisation and study of biofilm formation on GIC discs. (**A. Top view of the flow cell chamber B. Side view of the flow cell chamber).** The system is made up of three custom made flow cells made from Acetal Plastic as above containing three stainless steel inlets and three outlets at each end to create laminar flow. Medium flows through a three way adapter via a three channel peristaltic pump to the bubble trap which captures small air bubbles and prevents them from passing through the system and disrupting biofilm formation. Immediately after the bubble trap there are three inoculation ports which are used to inoculate the flow cells. Each flow cell contains a 30 mm x 50 mm insert containing a 10 mm round diameter recess to fit GIC discs for testing. The volume of each flow cell was 1.5 mL.

Biofilm culture of *S*. *mutans* Ingbritt on Fuji VII GIC material in flow cells was carried out using custom designed flow cells made from Acetal plastic with an adjustable screw down lid, a disposable coverslip and rubber gasket to seal the system. The flow cell measured 50 mm in length x 30 mm wide with a volume of 1.5 mL and contained a single circular cut out of 10 mm diameter to secure the GIC block for imaging. Each flow cell contained three inlet ports and efflux ports to ensure laminar flow was achieved in the system. Each system contained three individual flow cells connected to an inlet and an efflux manifold using a three way adapter. The inlet ports were connected to the medium reservoir via a bubble trap and peristaltic pump. The efflux ports were connected to an effluent bottle (**Fig 1**).

Flow cells and GIC discs were sterilised using gamma irradiation (4.1 kGy) and parts assembled aseptically in a Class II Biological Safety Cabinet (Contamination Control Laboratories Pty Ltd). Sterile 25% ASM was pumped through the system via a peristaltic pump for one min to coat the flow cells and remove air bubbles from the system. Each flow cell was inoculated with approximately 5 x 10^7^ *S*. *mutans* cells (1 x 10^7^ cells/mL) via a three way stop cock connected immediately preceding each flow cell. The flow cells were incubated for 1 h at 37°C prior to the commencement of a constant flow of 25% ASM through each cell at a rate of 5 mL h^-1^ for 16 h. The cells were then stained with BacLight Live/Dead stain prepared as per manufacturer’s instructions for 15 min and imaged using confocal laser scanning microscopy.

### Flow cell biofilm assay testing the effects of CPP-ACP challenge on established biofilms

Biofilm culture of *S*. *mutans* in flow cells was conducted similarly to Wen *et al*. [28] with several modifications. A 3-channel flow cell system (Stovall Life Science, Greensboro, North Carolina, USA) was modified with stopcocks for inoculation, testing and staining of the bacterial biofilms. All parts were assembled and 0.5% sodium hypochlorite was pumped in and left to stand overnight. Sterile water (200 mL) was then used to flush the system prior to the addition of 25% ASM.

Each channel of the system was inoculated with 1 mL of an exponentially growing *S*. *mutans* culture diluted to a cell density of 1×10^7^ cells/mL. The system was incubated for 1 h to allow bacterial cell attachment prior to constant flow of 12 mL h^-1^ of 25% ASM. After 16 h, 1 mL of 1% w/v CPP-ACP (pH 8.0), 0.5% CPP (pH 8.0) or sterile water (pH 8.0; positive control) was slowly injected into each channel of the system and incubated for 10 min, followed by another 10 min flow of 25% ASM. BacLight LIVE/DEAD stain prepared as per manufacturer’s instructions was then used to stain the biofilm *in situ* for 15 min.

CPP-ACP complex was prepared as described previously [29] and the complex was desalted using reversed-phase HPLC to yield CPP free of calcium and phosphate ions. Briefly the CPP-ACP complex was dissolved in 50 mM EDTA, pH 4.5 and injected onto a C18 column equilibrated with 0.1% trifluoroacetic acid (TFA). After removal of the unbound salts with 0.1% TFA (v/v), the bound CPP were eluted with 0.1% TFA (v/v) 80% acetonitrile (v/v). The collected CPP were lyophilized. The CPP and CPP-ACP were dissolved in deionised water and the pH adjusted to 8.0 before use.

### Confocal microscopy imaging

Confocal laser scanning microscopy (CLSM) was carried out on an LSM Meta 510 Confocal Microscope with an inverted stage (Carl-Zeiss, Oberkochen, Germany) essentially as described previously [30] with minor modifications. To determine reproducibility across the biofilm five images at random positions were obtained at wavelengths of 488 nm and 568 nm for each channel and three biological replicates were used. The images were 1024 x 1024 pixels (0.14 μm per pixel) with each frame at 142.72 μm (x) x 142.72 μm (y) for flow cell assays using GIC and (0.352 μm per pixel) with each frame 359.65 μm (x) x 359.65 μm (y) for the static assays using GIC. All CLSM images were analyzed using COMSTAT software [31].

### Statistical analysis

The biometric data obtained from the COMSTAT program were initially examined to determine if they were normally distributed. If the data were not normally distributed, as was the case with the aqueous CPP and CPP-ACP treatments the Kruskal-Wallis test and a Mann-Whitney U Wilcoxon rank sum test with a Bonferroni correction for type 1 error were used. Paired t tests were used to compare the GIC with CPP-ACP vs GIC without the complex for the flow cell experiments.

## Results

### Effects of CPP-ACP incorporation into GIC on the colonisation and establishment of *S*. *mutans* biofilms in a static assay

**Fig 2:**
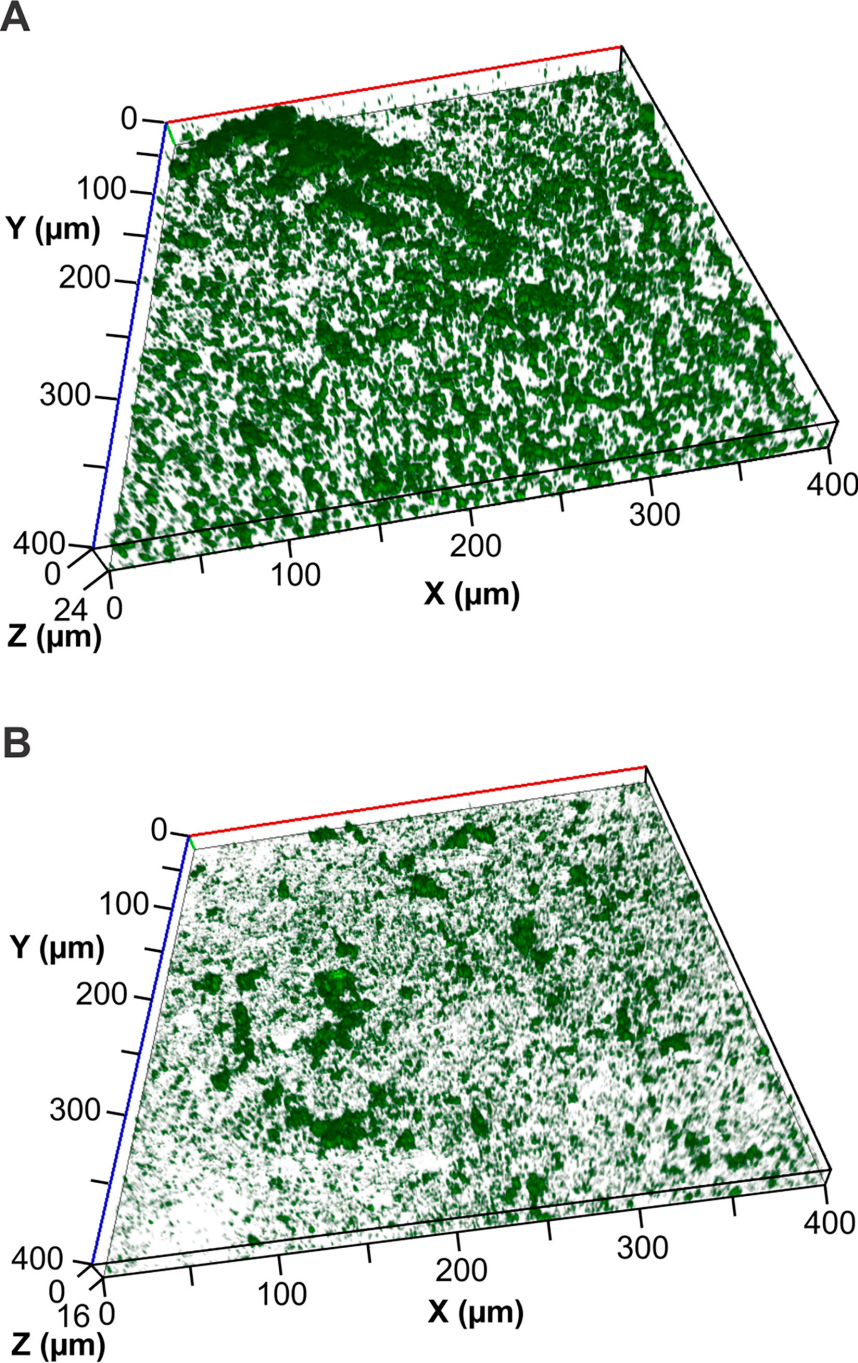
Representative images of *S*. *mutans* biofilm formation on Fuji VII GIC (A) and Fuji VII EP GIC (B) after 16 h growth in a static biofilm model.

Fuji VII and Fuji VII EP GIC blocks were placed into tissue culture wells containing 25% ASM and inoculated with *S*. *mutans*. After 16 h incubation *S*. *mutans* had formed extensive, structured biofilms on the Fuji VII material with a maximum thickness of 21.56 μm as determined by confocal microscopy image analysis (**Table 1 and Fig 2**). The inclusion of 3% CPP-ACP in Fuji VII EP significantly reduced the biovolume of the *S*. *mutans* biofilm by 50% relative to the Fuji VII control and average thickness of the biofilm was reduced by 66% (**Table 1 and Fig 2**). The structure of the biofilms formed on Fuji VII EP were significantly altered relative to Fuji VII with an increase to the surface area to volume ratio from 2.27 ± 0.45 to 3.24 ± 0.63, indicating a more open structure of the biofilms when CPP-ACP was present (**Fig 2**). There was no significant effect on the proportion of the substratum surface that was colonised by the bacterium.

**Table 1:** Biometric parameters of 16 h static *S*. *mutans* biofilms cultured using Fuji VII or Fuji VII EP as the substratum. Values are the mean and standard deviation of three biological replicates.

	Fuji VII GIC (no CPP-ACP)	Fuji VII EP (3% CPP-ACP)	% Change
Biovolume (μm^3^/μm^2^)	1.53 ± 0.33	0.76 ± 0.23*	-50%
Average thickness (μm)	1.88 ± 0.61	0.65 ± 0.11*	-66%
Maximum thickness (μm)	21.56 ± 8.94	17.16 ± 3.78	-19%
Surface area: Volume ratio	2.27 ± 0.45	3.24 ± 0.63*	+70%
Proportion of the substratum colonised (%)	14.92 ± 5.07	12.85 ± 4.50	-14%

* Significantly different from Fuji VII GIC p < 0.05

### Effects of Fuji VII EP on the colonisation and establishment of *S*. *mutans* biofilms in a flow cell

In order to better replicate the conditions observed *in situ* where exposed enamel is subjected to a constant flow of saliva containing fresh nutrients and imposing shear forces on biofilms, a custom made flow cell system was designed. This is the first time such a design, capable of imaging GIC discs undisturbed in real time has been reported in the literature. The system is similar in composition to some commercial systems with several improvements as it is composed of a re-useable flow cell with silicone rubber gasket seal and removable insert to accommodate the GIC blocks without disturbing the flow properties of the system (**Fig 1**).

**Fig 3:**
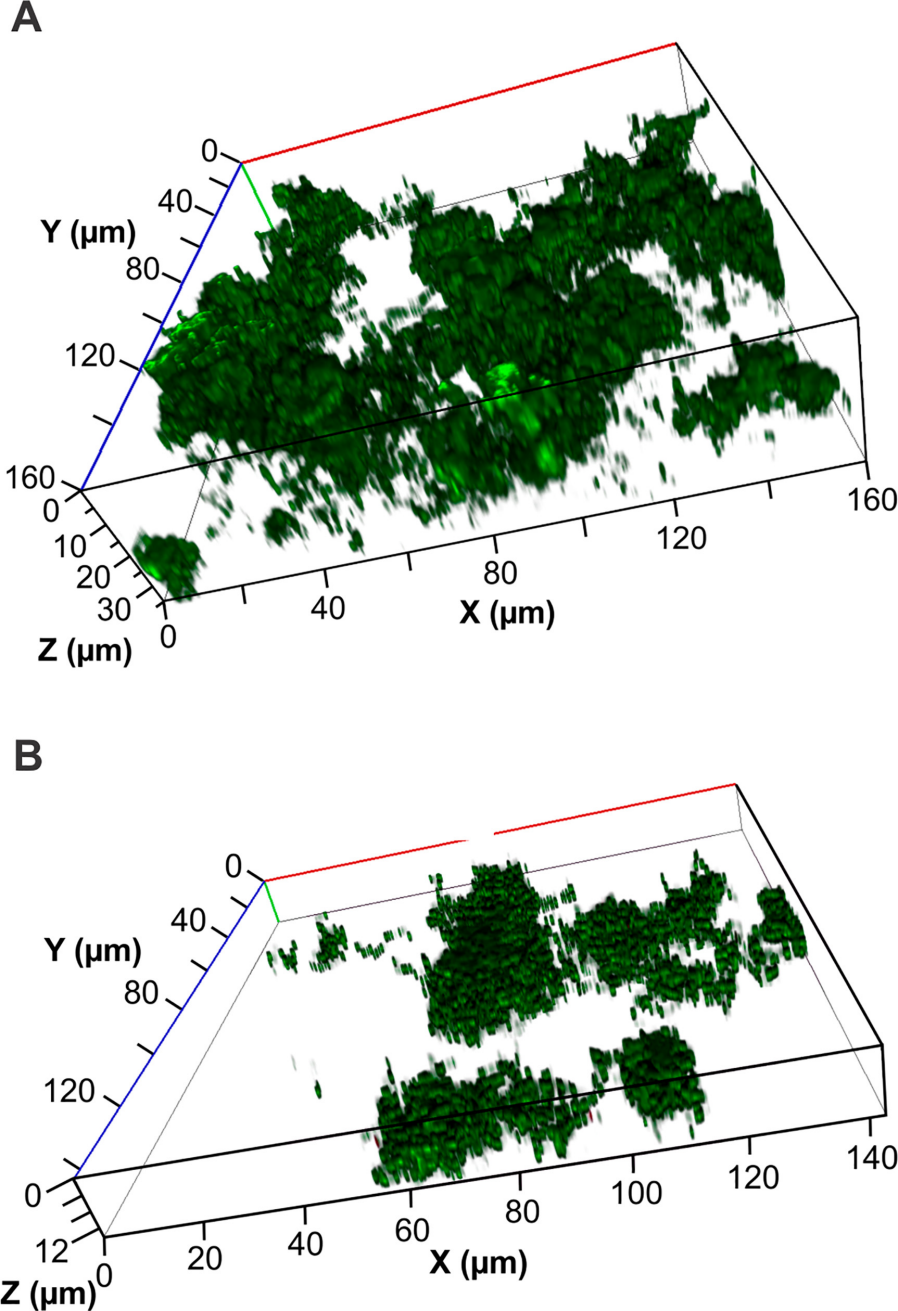
Representative images of *S*. *mutans* biofilm formation on Fuji VII GIC (A) and Fuji VII EP GIC (B) after 16 h growth in a flow cell system.

Biofilms formed on Fuji VII GIC in the flow cell system were significantly more dense compared with biofilms formed on the same material in the static system (**Figs 2 and 3**). There was a nearly two-fold increase in biovolume, a four-fold increase in average thickness, yet there was a four-fold decrease in the proportion of the substratum colonised by bacteria in the flow cell relative to the static assay (**Tables 1 and 2**). These differences clearly show the effects of shear force and a constant flow of nutrients, resulting in denser, more tenacious biofilms in the flow cells.

**Table 2:** Biometric parameters of 16 h flow cell *S*. *mutans* biofilms cultured using Fuji VII or Fuji VII EP as the substratum. Values are the mean and standard deviation of three biological replicates.

	Fuji VII GIC (no CPP-ACP)	Fuji VII EP (containing CPP-ACP)	% change
Biovolume (μm^3^/μm^2^)	2.74 ± 0.43	1.17 ± 0.03*	-57%
Average thickness (μm)	7.72 ± 2.11	1.45 ± 0.25*	-81%
Maximum thickness (μm)	37.07 ± 12.69	13.2 ± 1.83*	-64%
Surface area: Volume ratio	2.97 ± 0.53	2.45 ± 0.57	-18%
Proportion of the substratum colonised (%)	3.98 ± 1.23	6.05 ± 2.55	+34%

* Significantly different from Fuji VII GIC p < 0.05

After 16 h of incubation *S*. *mutans* formed highly structured biofilms on the GIC substratum with a biovolume of 2.74 μm^3^/μm^2^ (**Table 2 and Fig 3**). The incorporation of 3% CPP-ACP into the GIC caused significant reductions in the biovolume, average thickness and maximum thickness of the *S*. *mutans* biofilms formed in the flow cells (**Table 2**). There was no significant effect on the proportion of the substratum that was colonised by bacteria or the surface to volume ratio of the biofilms (**Table 2**).

### Effects of aqueous CPP-ACP treatment on established *S*. *mutans* biofilms in a flow cell

To examine the effects of CPP-ACP on established *S*. *mutans* biofilms, a flow cell system with a glass substratum was utilised. Biofilms were propagated within this system for the same time period as the previous experiment and then exposed to a single 10 min treatment of 1% CPP-ACP or 0.5% CPP. As approximately half the weight of CPP-ACP is CPP-stabilized amorphous calcium phosphate the 0.5% CPP treatment enabled the determination of the contribution of the CPP component to the effects seen with 1% CPP-ACP. *S*. *mutans* biofilms cultured in these flow cells that utilised the flow cell material as the substratum had a significantly higher biovolume than those cultured on GIC under similar conditions (**Tables 2 and 3**). The increase in biovolume was largely due to a significant increase in the proportion of the substratum colonised by *S*. *mutans* and the structure of the biofilm that displayed a significantly lower surface area to volume ratio of the biofilms cultured without GIC as the substratum (**Tables 2 and 3**). There was no significant difference in the average thickness of the biofilms. As this indicated an inhibitory effect of GIC itself on *S*. *mutans* biofilm development the effects of aqueous CPP-ACP on established *S*. *mutans* biofilms was determined without using GIC as the substratum.

**Table 3:** Effect of a single CPP-ACP treatment on an established 16 h *S*. *mutans* biofilm cultured in a 3-channel flow cell system as determined using COMSTAT analysis of CLSM images. Values are the mean and standard deviation of three biological replicates. The percentage change relative to the control is shown in brackets.

	Control (no CPP-ACP)	0.5% CPP	1% CPP-ACP
Biovolume (μm^3^/μm^2^)	4.22 ± 1.18	3.61 ± 1.11 (-14.4%)	1.76 ± 0.68* (-58.4%)
Average thickness (μm)	7.63 ± 2.29	6.10 ± 1.92 (-20.1%)	3.23 ± 1.53* (-57.7%)
Maximum thickness (μm)	28.4 ± 4.08	28.67 ± 4.32 (+0.9%)	24.4 ± 3.94 (-14.08%)
Surface area: Volume ratio	1.98 ± 0.38	2.07 ± 0.47 (+4.2%)	2.45 ± 0.34 (+19.2%)
Proportion of the substratum colonised (%)	13.19 ± 6.52	15.23 ± 5.09 (+13.4%)	9.89 ± 2.90 (-25%)

* Significantly different from control p < 0.05

**Fig 4:**
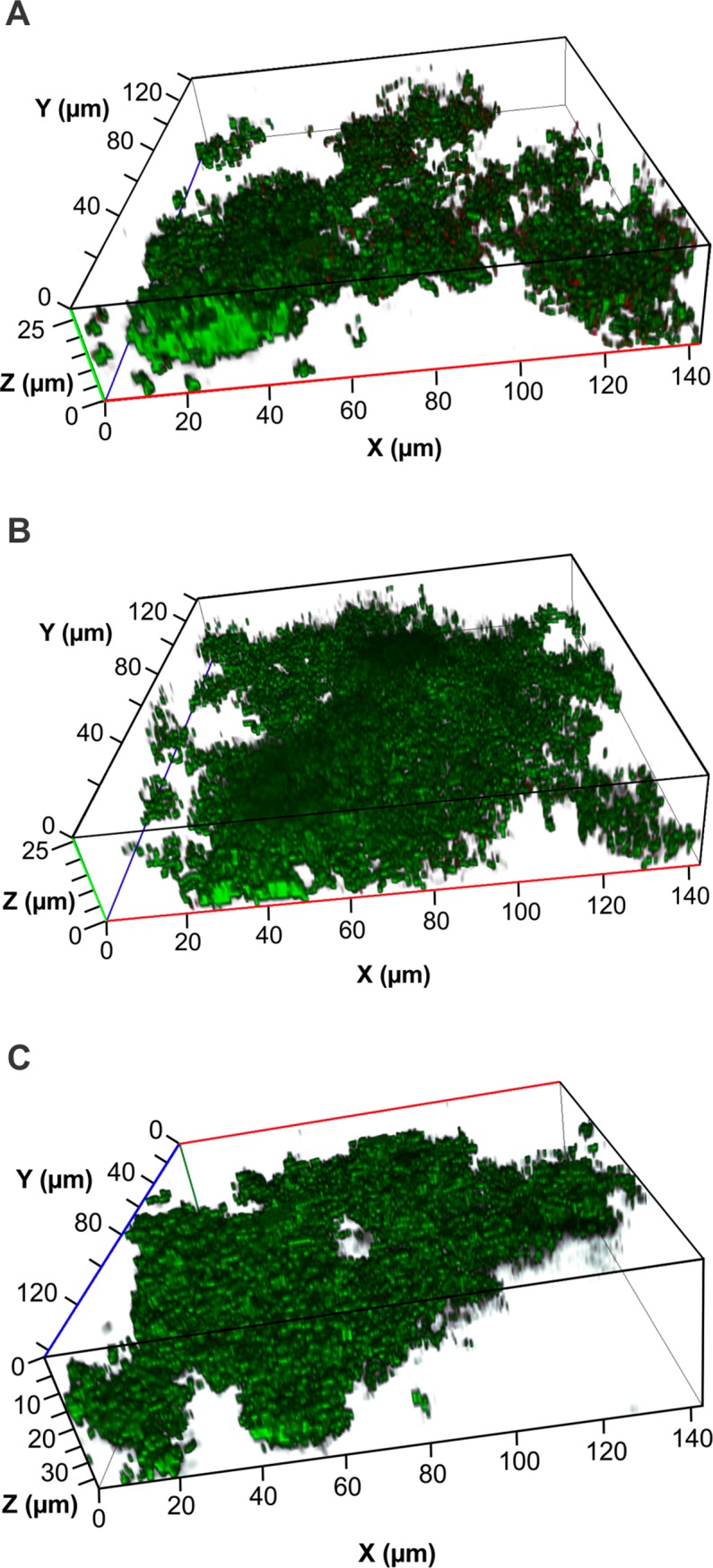
Representative images of the effect of a ten minute treatment of 1.0% CPP-ACP (A) and 0.5% CPP (B) on 16 h *S*. *mutans* biofilms (C) cultured in a flow cell.

A single ten min treatment with 1% CPP-ACP resulted in significant 58% decreases to both the biovolume of the biofilm and the average thickness (**Table 3 and Fig 4**). There was no significant change to the surface area:volume ratio of the biofilm on treatment with CPP-ACP. Treatment with 0.5% CPP had no significant effect on any of the biometric parameters of the *S*. *mutans* biofilm (**Table 3 and Fig 4**). A single 10 min treatment of the established 16 h *S*. *mutans* biofilms with 2% CPP-ACP resulted in such severe disruption of the biofilm that they could not be visualised and quantified by confocal imaging (data not shown).

## Discussion

The use of amalgam in dental restorations has decreased over the last decade due to its potential health effects and biopersistence [32–34]. This has resulted in the increased use of alternative restorative materials including the relatively recently developed GICs. GICs have often been referred to as “smart materials” as they have a number of properties which may be altered in a controlled fashion in response to stimuli. These include ion exchange and recharge between the GIC and external environment, chemical adhesion to tooth enamel and dentin and a thermal expansion coefficient which is similar to teeth [35–37]. GICs also allow the incorporation of bioactive molecules including fluoride, strontium, calcium and phosphate that can be released to extend the influence of the material beyond its boundaries [27]. Previous studies have shown that GICs are capable of remineralising carious tissue due to their high content of apatite forming ions and can prevent secondary caries lesions through ion release and chemical bonding properties with tooth enamel [37]. Al Zraikat *et al*. [26] demonstrated that incorporation of 3% CPP-ACP into GIC resulted in an enhanced ability to inhibit enamel demineralization induced by exposure to lactic acid buffered at pH 4.8. This was attributed to the release of calcium, phosphate and fluoride in a bioavailable form mediated by CPP-ACP.

Bacterial biofilm formation on GICs can lead to increased roughness and surface deterioration which can further enhance microbial biofilm development [38]. This can eventually lead to the development of secondary caries around or below the GIC restoration [39, 40]. GICs have recently been used as delivery vehicles for specific antimicrobials and these materials were shown to inhibit *S*. *mutans* growth [41]. However little work has been published directly indicating antibiofilm activity of GICs containing these types of compounds.

We used two distinct biofilm assays to determine the effect of incorporation of CPP-ACP into GIC on *S*. *mutans* colonisation of the surface and biofilm development. The static biofilm assay is more reflective of protected, stagnant sites, such as interproximal areas that have limited saliva flow and low shear forces, whereas the flow cell assays are representative of more exposed enamel sites that have a higher saliva flow. Both assays used the same mucin-rich, artificial saliva growth medium. *S*. *mutans* biofilms cultured in the flow cell were significantly more dense than those cultured in the static assay for the same time period (**Tables 1 and 2**). This is reflective of the continuous supply of nutrients in the flow cell and may also be a response to the shear forces imposed in the flow cell model. A similar inhibition of *S*. *mutans* biofilm development by incorporation of 3% CPP-ACP was obtained with both assays showing significant reductions of over 50% in the biovolume of the 16 h *S*. *mutans* biofilms (**Tables 1 and 2**). This indicates that shear forces do not play a role in the mechanism of biofilm inhibition. CPP-ACP incorporation into the GIC also did not significantly reduce the proportion of the surface colonised by *S*. *mutans* indicating that the biofilm inhibitory effect is not related to attachment of the bacterial cells to the substratum.

In this study we showed that *S*. *mutans* biofilms formed on a glass substratum after 16 h had a significantly higher biovolume than those formed on GIC using the same growth medium with similar flow conditions and shear forces (**Tables 2 and 3**). This is consistent with recent studies that have indicated that GICs have an inherent inhibitory effect on bacterial growth, possibly due to the release of fluoride or other ions [42]. The incorporation of 3% CPP-ACP into the GIC further significantly inhibited *S*. *mutans* biofilm development by over 50% (**Table 2**). This is consistent with a recent *in situ* study that demonstrated a delay in plaque (biofilm) formation on Germanium surfaces treated with CPP-ACP in comparison to those without treatment [43]. In our study the proportion of the substratum that was colonised by *S*. *mutans* was not significantly different in the CPP-ACP containing GIC compared to the GIC alone, indicating that initial colonisation of the surface by the bacterium was not inhibited. This suggests that it was biofilm development that was inhibited by CPP-ACP that had diffused from the GIC [27]. CPP-ACP has been shown to bind to *S*. *mutans* and the intercellular plaque matrix [18, 19] and analysis of plaque samples demonstrated CPP-ACP nanocomplexes to be localized in plaque on the surfaces of bacterial cells [14]. The binding of the CPP-ACP nanocomplexes to the surface macromolecules of S. *mutans* is likely to be mediated by calcium cross-linking of cell surface phosphate moieties and by hydrophobic and hydrogen-bond mediated interactions [14, 44]. This binding by CPP-ACP would mask cell surface macromolecules essential for cell to cell adhesion and biofilm development.

To determine the effects of aqueous CPP-ACP treatment on established *S*. *mutans* biofilms we used the glass substratum to avoid the confounding effects of the inherent antibiofilm activity of the GIC. A single ten min treatment with 1% aqueous CPP-ACP resulted in a 58% decrease in both biovolume and average biofilm thickness (**Table 3**). There was no obvious increase in the number of dead cells in these analyses indicating that CPP-ACP did not have direct bactericidal activity. The disruption of these established biofilms was not due to the CPP component of the CPP-ACP as treatment with a 0.5% CPP had no significant effect on the biometric parameters of the established *S*. *mutans* biofilms (**Table 3**). Increasing the added CPP-ACP to 2% effectively eradicated the established *S*. *mutans* biofilm confirming that the intact nanocomplexes were essential for the dose-dependent disruptive effect on the established biofilm.

In this study we have shown that incorporation of CPP-ACP into GIC significantly reduced biofilm development and that treatment with aqueous CPP-ACP disrupted established biofilms. In the clinical setting the use of CPP-ACP containing GIC coupled with regular aqueous CPP-ACP treatment may reduce bacterial biofilm establishment and development and therefore may reduce bacteria-associated damage to the GIC surface and the development of caries.
